# Community mobilization, empowerment and HIV prevention among female sex workers in south India

**DOI:** 10.1186/1471-2458-13-234

**Published:** 2013-03-16

**Authors:** Andrea K Blanchard, Haranahalli Lakkappa Mohan, Maryam Shahmanesh, Ravi Prakash, Shajy Isac, Banadakoppa Manjappa Ramesh, Parinita Bhattacharjee, Vandana Gurnani, Stephen Moses, James F Blanchard

**Affiliations:** 1 Department of Community Health Sciences, University of Manitoba, S113 Medical Service Building, 750 Bannatyne Avenue, Winnipeg, MB, R3E 0W3, Canada; 2 Karnataka Health Promotion Trust, Bangalore, India; 3 Research Department of Infection and Population Health, University College London, London, United Kingdom; 4 Resident Commissioner, Karnataka, New Delhi, India

## Abstract

**Background:**

While community mobilization has been widely endorsed as an important component of HIV prevention among vulnerable populations such as female sex workers (FSWs), there is uncertainty as to the mechanism through which it impacts upon HIV risk. We explored the hypothesis that individual and collective empowerment of FSW is an outcome of community mobilization, and we examined the means through which HIV risk and vulnerability reduction as well as personal and social transformation are achieved.

**Methods:**

This study was conducted in five districts in south India, where community mobilization programs are implemented as part of the *Avahan* program (India AIDS Initiative) of the Bill & Melinda Gates Foundation. We used a theoretically derived “integrated empowerment framework” to conduct a secondary analysis of a representative behavioural tracking survey conducted among 1,750 FSWs. We explored the associations between involvement with community mobilization programs, self-reported empowerment (defined as three domains including *power within* to represent self-esteem and confidence, *power with* as a measure of collective identity and solidarity, and *power over* as access to social entitlements, which were created using Principal Components analysis), and outcomes of HIV risk reduction and social transformation.

**Results:**

In multivariate analysis, we found that engagement with HIV programs and community mobilization activities was associated with the domains of empowerment. *Power within* and *power with* were positively associated with more program contact (p < .01 and p < .001 respectively). These measures of empowerment were also associated with outcomes of “personal transformation” in terms of self-efficacy for condom and health service use (p < .001). Collective empowerment (*power with others*) was most strongly associated with “social transformation” variables including higher autonomy and reduced violence and coercion, particularly in districts with programs of longer duration (p < .05). Condom use with clients was associated with *power with others* (p < .001), while *power within* was associated with more condom use with regular partners (p < .01) and higher service utilization (p < .05).

**Conclusion:**

These findings support the hypothesis that community mobilization has benefits for empowering FSWs both individually and collectively. HIV prevention is strengthened by improving their ability to address different psycho-social and community-level sources of their vulnerability. Future challenges include the need to develop social, political and legal contexts that support community mobilization of FSWs, and to prospectively measure the impact of combined community-level interventions on measures of empowerment as a means to HIV prevention.

## Background

There are an estimated two and half million people living with HIV in India who will ultimately require lifelong treatment with antiretroviral therapy (ART). This large burden of infection makes tackling the HIV epidemic in India a health priority. HIV preventive interventions targeting female sex workers (FSWs) are considered to be pivotal to HIV prevention efforts in India [1,2]. The optimism engendered by the addition of an ever expanding arsenal of individual level HIV prevention technologies, including male circumcision, antiretroviral treatment as prevention, pre-exposure prophylaxis and potentially a topical microbicide, should be tempered by the growing evidence that psychosocial and community-level processes underlie an individual’s ability to adopt safer sexual behaviours and access new HIV prevention technologies [2-5]. There is therefore an even greater urgency to describe and understand the mechanisms through which interventions impact on the risk environment and facilitate an individual’s ability to adopt and accept HIV prevention technologies.

Community mobilization of FSWs has been endorsed as one of the structural interventions that improve the risk environment, and has thus become a central tenet of HIV prevention [6-11]. The effectiveness of community mobilization in addressing health and social issues of poor and marginalized populations is largely explained through “empowerment” [12-15]. Although there have been successful examples of community mobilization and empowerment of FSWs, particularly in the Indian context, there remains considerable uncertainty around the mechanism through which community mobilization may impact upon FSWs’ HIV risk [8,14,16-18]. This creates barriers to replication of successful interventions, and also makes it difficult to monitor and evaluate the impact of community mobilization over time.

In this paper we outline an “integrated empowerment framework” based on theoretical and program literature, and use it to empirically examine the pathways between empowerment, both as an outcome of community mobilization strategies, and as a means to promote social transformation and HIV risk reduction amongst FSWs in south India.

## Methods

### Integrated empowerment framework for FSWs

**Figure 1:**
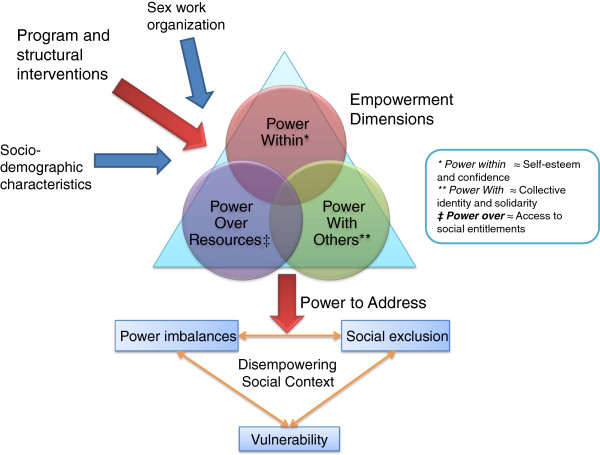
Integrated empowerment framework.

Based on the literature and on theoretical considerations, with particular influences from the work of Naila Kabeer, Amartya Sen, and Anisur Rahman, we developed an integrated empowerment framework for FSWs. A disadvantaged position of power, reinforced through stigma and social exclusion, has been found to contribute to the vulnerability of female sex workers to HIV in India and elsewhere [14,18,19]. Empowerment strategies are advocated as a mechanism whereby FSWs achieve the *power to* overcome this disadvantaged position and gain the agency to address their HIV vulnerability [13,20]. Sub-processes of *power to* can be seen to operate in three interrelated domains: *power within*, *power with (others*), and *power over (resources*) [13,21,22]. Programs have emphasized the importance of *power within,* as a means to develop FSWs’ self-esteem, confidence, and consciousness of the sources of their HIV vulnerability [14,18,23]. Programs have also emphasized the need for collective empowerment, or *power with others*, to effectively address power imbalances and to achieve social transformation [3,13,22-27]. Thus, programs have adopted community mobilization strategies that aim to develop a collective identity, trust, and mutual support as the basis of collective action [8,14,16]. In addition, domains of *power within* and *power with* must be complemented by the ability to exert *power over resources *[8,22,26,28]. Therefore, empowerment strategies have been used to increase FSWs’ access to social entitlements, financial credit and educational opportunities [8,14,29-31]. It has also been recognized that community mobilization strategies among FSWs must be complemented by structural interventions to “bring about comprehensive changes in the social, economic, legal and political structures that led to disempowerment in the first place” [25,32-35]. Other factors influencing women’s individual ability to benefit from empowerment programs include her socio-demographic characteristics (age, marital status, education level, caste status), and the type of sex work she practices (duration, number of clients, typology, etc.). Structural programs beyond community mobilization have been found to support community mobilization programs by creating an enabling environment wherein all three domains of empowerment are more attainable among FSWs [Bhattacharjee P, Prakash R, Pillai P, Isac S, Haranahalli M, Blanchard AK, Shahmanesh M, Blanchard JF, Moses S: WORLD BANK Special Issue: Understanding the role of peer group membership in reducing HIV-related risk and vulnerability among female sex workers in Karnataka, in preparation]. This integrated empowerment framework is articulated in Figure 1.

### Study design and context

This study was conducted in the context of community mobilization programs implemented by the Karnataka Health Promotion Trust (KHPT) and the University of Manitoba as part of *Avahan*, the India AIDS initiative of the Bill & Melinda Gates Foundation. We focused on five districts where data were collected through cross-sectional behavioural tracking surveys (BTS) conducted in 2010: Belgaum, Gulbarga, Gadag and Dharwad districts in Karnataka, and Solapur in Maharashtra. These districts were purposively chosen to represent different socio-cultural regions. Details of the programmatic context have been described previously [36]. KHPT’s programs were initiated in each district between 2002 and 2008, and involved comprehensive outreach, education and other services for FSWs to reduce the risk of transmission of HIV and other sexually transmitted infections (STIs), by promoting behaviour change and improved access to health services. In addition, the program implemented and scaled up community-based mobilization activities focusing on three main areas to empower FSWs. First, the program addressed *power within* by aiming to build individual capabilities to foster enhanced self-esteem, confidence and agency among individual FSWs. It engaged the FSW community in proactively analyzing the sex trade, developing a more positive perception of their identity as sex workers, and an awareness of the sources of their HIV risk and vulnerability. Secondly, the program aimed to foster the development of *power with* through the formation of collective identities, processes and capabilities to allow FSWs to address their immediate needs, including dealing with crises and improving the delivery of programs and services for HIV prevention. The program also encouraged the collective challenging of socio-cultural and structural factors underlying their risk and vulnerability. Thirdly, the program aimed to improve *power over resources* by providing access to social entitlements from the state (ration cards, voter ID and bank accounts) as well as micro-loans for self-employment opportunities. Finally, beyond community mobilization the KHPT programs have tried to sensitize a range of stakeholders in and beyond the community-level to address factors in the macro-level social environment that pose structural barriers to empowerment among FSWs. The BTS was used to gather detailed information on demographic characteristics, sex-work practice, program involvement, community mobilization and empowerment indicators, including experience of violence, condom use and service utilization.

### Sample size and study participants

It was estimated that a sample size of 350 FSWs in each district, or 1,750 in total, was required for the BTS. Sample size calculations were undertaken to detect a 15% change in the outcome of condom use between this initial round and planned subsequent survey rounds, with 90% power and alpha error of 5%, assuming a baseline value of 50% for condom use as a standard outcome. A probability-based sampling method was used to select women from various geographical sites (rural and urban) and different sex work typologies, to be representative of the total population of FSWs in each district. Conventional cluster (location) sampling was used in places where the population of FSWs was fairly fixed, and time-location clusters (TLCs) were sampled for public places where FSWs solicited, with an equal probability of selection from an exhaustive sampling frame (established through mapping of the operational typologies) [9].

### Data collection and management

Data were collected by trained researchers using interviewer-administered culturally sensitive and context-specific questionnaires, adapted from previous surveys and conducted in *Kannada* or another local language. Data from the BTS were entered using CSpro version 4 (U.S. Census Bureau, USA). Empowerment variables were created using weighted composite indicators for each of the three empowerment domains separately. These composite empowerment indicators were first created by entering relevant questions for each domain into principal component analysis (PCA). The variables used were recoded to reflect positive (+1 and + 2) or negative (−1 and −2) levels of empowerment. The values of + 2 and +1 were used when respondents stated that they “strongly agree” or “agree” respectively to questions that reflect higher levels of empowerment in each of the domains. Similarly, -2 and −1 were used when respondents reported “strongly disagree” or “disagree” to questions in each sub-domain in a way that represented a sense of low empowerment or disempowerment. PCA gives each question a component loading, which describes linear combinations of the variables that contain most of the variation in the data. This helps to reduce the data to a smaller set of components [37]. Variables were grouped into a given component if the variable’s component loading was more than 0.4 for that component and less than 0.4 for the other components [38]. For *power with* and *power within* there were two components each and for *power over resources* there was one component (see Table 1 for variables and component loadings). For only *power with,* there were items with component loadings lower than 0.4 that were thus excluded from analysis due to redundancy (“In general, your colleagues in the area where you work only worry about themselves,” and “In general, the people you work with are always arguing among each other”). The variables that were retained in each empowerment domain’s PCA were weighted by multiplying their component loading by each individual’s original value for that variable. These products were summed and normalized to give each woman who participated in the survey a composite value to reflect women’s individual levels of *power within*, *power with* and *power over*.

**Table 1:** Questionnaire items included in the composite indicators of the empowerment domains derived from principal components analyses

**“Power within”**	**Component loadings from PCA**		**“Power with”**	**Component loadings from PCA**		**“Power over resources”**	**Component loadings from PCA**
**Questions**	**Component 1**	**Component 2**	**Questions**	**Component 1**	**Component 2**	**Questions**	**Component 1**
I do not feel ashamed to say I am a SW in a meeting with other SWs	.905*	-.068	How confident are you that sex workers can work together to keep each other safe from harm?	.137	.607*	Possession of a voter ID	.931*
I do not feel ashamed to say I am a SW in a meeting with social/health workers	.994*	-.052	How confident are you that sex workers can work together to increase the use of condom?	-.060	.676*	Possession of a bank account	.471*
I do not feel ashamed to go on my own to NGO/CBOs	1.012*	-.084	How confident are you that sex workers can work together to speak up for your rights?	-.086	.882*	Possession of a ration card	.957*
How confident do you feel in giving advice to your fellow SWs, neighbours, or friends?	.224	.939*	How confident are you that sex workers can work together to improve your lives?	-.079	.845*		
How confident do you feel speaking your opinion in a large group of people?	-.016	.793*	Can you count on your colleagues if you need to borrow money?	.608*	-.032		
			Can you count on your colleagues to accompany you to the doctor or hospital?	.601*	.016		
			Can you count on your colleagues if you need to talk about your problems?	.575*	.105		
			Can you count on your colleagues if you need advice?	.670*	.089		
			Can you count on your colleagues if you need somewhere to stay?	.673*	.037		
			Can you count on your colleagues to help with a violent or difficult client?	.601*	.011		
			Can you count on your colleagues to support the use of condom?	.700*	-.062		
			The group of women with whom you work is an integrated group	.636*	.074		
			You can trust the majority of the people working in your area	.605*	.069		
			In general, the people you work with get along well	.758*	-.015		

*Asterisks signify that the question loads onto that component since the component loading is >0.4 (see Methods).

Proximate measures of social and personal transformation were also created using Principal Components Analysis. There were five components derived using the same method described above, including: autonomy and violence or abuse from more powerful groups for “social transformation”; and self-efficacy for condom use with clients, self-efficacy for condom use with regular partners, and self-efficacy for service utilization to reflect “personal transformation”. Questions that had many “no responses” were not used, including “Do you need permission to conceive a child?” and “Do you need permission to use family planning methods?”, as well as “Stopped carrying condoms for fear of police” and “Number of times given something to police to avoid trouble” Finally, “HIV program behavioural outcomes” were also analyzed using the original measures of HIV risk in the BTS, including reported levels of condom use on average and at last sex, and service utilization in the last six months. All outcome variables were recoded into binary outcomes by splitting the original values into “high” and “low” categories for autonomy and self-efficacy, condom use, and service utilization, and “ever” or “never” for physical and sexual violence as well as being arrested.

### Statistical analysis

Quantitative analysis was conducted using IBM SPSS Statistics, Version 19.0 (IBM, Armonk, New York). Demographic characteristics of the study population were assessed by comparing means and through analysis of cross-tabulated data as shown in Table 2. For multivariate analysis, a two-step pathway analysis was used to provide interpretable results that could accommodate variables with linear or curvilinear relationships. For the analysis of association between program involvement and empowerment levels, generalized linear models were used to obtain adjusted means for each empowerment domain as an outcome in relation to program intervention variables as the question predictors while controlling for socio-demographic characteristics and sex work practice variables. Adjusted means and significance levels from this analysis are presented in Table 3.

**Table 2:** Description of key socio-demographic and sex work characteristics, program intervention variables, and contextual and HIV risk factors in a behavioural tracking survey of 1750 female sex workers in south India

**Characteristic**	**Belgaum**	**Gulbarga**	**Gadag**	**Dharwad**	**Solapur**	**All districts**
	**n = 383**	**n = 374**	**n = 288**	**n = 385**	**n = 361**	**N = 1751**
**Socio-demographic variables**						
*Age (mean, SD)*	31.62 (6.45)	32.08 (7.14)	33.10 (6.98)	33.55 (7.19)	30.68 (7.33)	32.15 (7.08)
*Unable to read or write*	74.6%	79.1%	79.4%	76.3%	64.4%	74.8%
*Main partner status*						
No partner	23.8%	20.1%	27.4%	25.9%	9.7%	21.1%
Boyfriend (Not live-in or cohabiting)	23.2%	17.9%	13.9%	11.9%	46.0%	23.0%
Boyfriend (Live-in or cohabiting)	14.1%	21.4%	15.6%	16.9%	8.0%	15.2%
Husband	38.9%	40.6%	43.1%	45.3%	36.3%	40.7%
**Sex-work practice variables**						
*Start sex work aged less than 18*	11.3%	6.7%	6.6%	6.4%	12.9%	8.9%
*Duration in SW less than 2 years*	7.3%	10.4%	7.7%	18.6%	9.4%	10.7%
*Type of Sex Work*						
Street or other public place	52.8%	65.2%	71.4%	59.6%	50.1%	59.3%
Brothel	1.0%	1.6%	0.3%	0.0%	7.0%	2.1%
Home	9.7%	3.2%	7.7%	16.3%	16.0%	10.6%
Contacted by phone	35.2%	29.9%	20.2%	22.4%	10.4%	24.0%
*Client no. last seven working days*						
Less than 5	74.2%	64.3%	87.5%	62.2%	9.7%	58.5%
5-9	24.0%	30.9%	11.5%	31.4%	30.4%	26.2%
More than 10	1.8%	4.8%	1.0%	6.4%	59.9%	15.2%
**Program Intervention Variables**						
*Member of an FSW community based organization*	85.1%	77.5%	86.8%	70.9%	62.2%	76.2%
*Contacted by program more than one year ago*	91.7%	88.1%	93.0%	79.4%	57.3%	81.8%
**Contextual Risk Factors**						
*Ever been arrested*	6.5%	4.3%	3.1%	15.1%	37.2%	13.5%
*Police coercion over past 6 months (given anything to police to avoid trouble)*	13.7%	12.0%	12.2%	16.0%	34.6%	18.2%
*Experienced physical violence in the past 6 months*	12.8%	9.4%	16.0%	18.8%	52.9%	22.1%
*Experienced forced sex in the past 6 months*	10.7%	10.7%	12.2%	16.%	34.5%	16.9%
**HIV Risk Factors**						
*Always use condoms with clients*	99.3%	98.3%	99.4%	96.4%	87.1%	95.4%
*Always use condoms with regular clients*	96.9%	97.3%	94.1%	95.5%	82.9%	93.3%
*Always use condoms with regular (non-paying) male partner*	49.0%	47.8%	44.7%	36.1%	30.2%	41.2%

**Table 3:** Adjusted empowerment means for socio-demographic, sex work and program variables for a sample of 1750 female sex workers in south India

**Characteristic**	**All districts**			**Belgaum Gulbarga and & Gadag (high intensity)**			**Dharwad and & Solapur (low intensity)**		
	**Power within**	**Power with**	**Power over**	**Power within**	**Power with**	**Power over**	**Power within**	**Power with**	**Power over**
**Age**									
18-21 (ref)	-.15	-.08	-.61	.42	.11	-.46	-.97	-.47	-.90
22-25	-.09	-.20	-.56 ‡	.40	.002	-.44	-.76	-.55	-.80
26-30	-.10	-.07	-.18 ‡	.27	.08	-.03 **	-.56 *	-.33	-.46 *
31-35	-.04	-.14	.12 ‡	.32	.03	.30 ‡	-.52 *	-.38	-.22 **
36 +	.03	-.03	.39 ‡	.36	.18	.60 ‡	-.40 *	-.36	.04 ‡
**District**									
Belgaum (ref)	.28	.26	.17	.40	.20	.16			
Gulbarga	.06‡	.11	-.13 ‡	.15‡	.05 ‡	-.14 **			
Gadag	.42‡	.04	-.01‡	.51 **	-.01**	-.03 ‡			
Dharwad	-.15‡	-.05	-.19 ‡				-.16	.06	-.20
Solapur	-.96‡	-.87	-.68 ‡				−1.13 ‡	-.89 ‡	-.73 ‡
**Type of sex work**									
Street based (ref)	-.04	-.16	-.20	.32	.14	-.02	-.61	-.58	-.52
Home	-.07**	-.18	-.16	.17	-.03 **	-.03	-.55	-.50	-.45
Contacted by phone	-.18	-.08	-.17	.18 **	.15	-.02	-.75	-.35 *	-.43
Brothel, vehicle, bar/nightclub^a.^	.02	.01	-.14	.74 ‡	.07 *	.05	-.66	-.25 *	-.48
**Client no. past 7 working days**									
< 5 (ref)	-.07	-.18	-.10	.44	.03	.07	-.81	-.53	-.41
5+	-.06	-.03*	-.23	.26 *	.13 *	-.08	-.47 **	-.30	-.52
**Total no. of sexual partners past 7 working days**									
<5 (ref)	.04	-.20	-.02	.39	.15	-.05	-.40	-.33	-.48
5+	-.18**	-.17	-.19**	.31	.01	.04	-.88 ‡	-.51	-.46
**Member of CBO**									
No (ref)	-.06	-.18	-.16	.34	.04	-.02	-.64	-.54	-.48
Yes	-.07	-.03**	-.17	.36	.12	.01	-.65	-.30 **	-.45
**Time since first program contact**									
Less than 6 months (ref)	-.30	-.42	-.12	.19	-.16	-.06	-.69	-.73	-.41
6-12 months ago	-.10*	-.15**	-.19	.33	.07	-.0003	-.62	-.44 **	-.51
1-2 years ago	.02‡	.04	-.20	.38	.14	.04	-.71	-.26 ‡	-.55
>2 years ago	.11‡	.12	-.14	.50 *	.27 *	.01	-.55	-.26 ‡	-.40
**Number of times contacted by peer educator in past 6 months**									
<10 (ref)	-.19	-.37	-.20	.14	-.11	.02	-.53	-.68	-.51
10-14	-.20	-.20**	-.11	.13	.02	-.09	-.73	-.66	-.28 **
15-19	-.06*	-.05	-.14	.35 *	.15 *	.05	-.61	-.61	-.50
20-24	.02**	.02	-.08	.42 **	.20 **	.06	-.34	-.42 *	-.30 *
25-29	-.09	-.07**	-.21	.38 *	-.08	.04	-.61	-.73	-.75
30 +	.12‡	.05	-.24	.69 ‡	.29 ‡	-.10	-.60	-.75	-.47

Adjusted means and p-values obtained from a generalized linear model analysis for each predictor variable with the Empowerment variables as the outcomes and all significant socio-economic and sex-work practice variables.

“Power within” represents a sense of individual self-esteem and confidence, “power with” reflects collective identity and solidarity, and “power over resources” reflects access to social entitlements.

a. The sex-work typology categories “brothel, vehicle, bar and night-club” were combined to create categories with more equal n-values. Age was split into categories based on equally sized quintiles.

* = p < .05, ** = p < .01, ‡ = p < .001.

Subsequently, binary logistic regression was used to assess associations between the empowerment domains and social transformation, personal transformation and HIV program behavioural outcomes while controlling for demographic, sex-work practice, and program intervention variables. Odds ratios and significance levels are provided in Table 4. The interpretation of odds ratios in Table 4 is such that with every one unit increase in *empowerment* level for a given domain, the odds of a high level of the outcome versus a low level is multiplied by the odds ratio. For example, for the odds ratio for autonomy in relation to *power within* in high intensity districts Belgaum, Gadag and Gulbarga was 1.22, which means that with every one unit increase in *power within* level, the odds of reporting a high autonomy level is 1.22-times higher than the odds of reporting a low autonomy level.

**Table 4:** The association between empowerment, personal and social transformation, and HIV program outcome variables, adjusted for background characteristics for a sample of 1750 female sex workers in south India

**Indicators**		**Social transformation variables**		**Personal transformation variables**			**HIV program outcome variables**				
		**Autonomy**	**Violence or abuse by more powerful groups**	**Self-efficacy for condom use with regular partner**	**Self-efficacy for condom use with clients**	**Self-efficacy for service utilization**	**Condom use at last sex with regular client**	**Frequency of condom use with regular clients**	**Condom use at last sex with regular partner**	**Frequency of condom use with regular partner**	**Number of times in last 6 months visited health clinic for health problems**
**All districts**	Power within	0.93	0.96	1.26‡	0.93	2.48‡	0.92	0.96	1.03	1.16	1.16*
	Power with	1.08	1.13	1.03	1.31‡	1.40‡	1.92‡	1.95‡	1.17*	1.20*	0.96
	Power over	1.07	1.06	1.07	0.97	1.01	0.61*	0.84	1.07	1.07	0.99
**Belgaum Gulbarga & Gadag (high intensity)**	Power within	0.87	0.89	1.27*	0.83	3.13‡	0.92	1.14	1.23	1.54**	1.05
	Power with	1.22*	1.34*	0.92	1.34**	1.72‡	2.56‡	2.27‡	1.09	1.06	1.00
	Power over	1.06	1.00	1.05	0.95	1.00	0.89	1.24	0.97	0.95	0.92
**Dharwad & Solapur (low intensity)**	Power within	1.04	1.02	1.28*	1.22	1.70‡	0.90	0.87	1.00	1.09	1.26*
	Power with	1.01	1.04	1.08	1.26*	1.19	1.65**	1.80‡	1.20	1.21	1.04
	Power over	1.14	1.17	1.06	1.25	0.98	0.38**	0.55**	1.19	1.22	1.08

Odd Ratios and p-values for each Empowerment domain were obtained using binary logistic regression for each outcome variable with all significant socio-demographic and sex work practice variables as covariates.

“Power within” represents a sense of individual self-esteem and confidence, “power with” reflects collective identity and solidarity, and “power over resources” reflects access to social entitlements.

* = p < .05, ** = p < .01, ‡ = p < .001.

The associations were calculated for all women and then disaggregated by intensity of the community mobilization efforts, i.e. between those with higher intensity and longer duration of community mobilization (Belgaum, Gulbarga and Gadag) and those with lower intensity and shorter duration of community mobilization (Dharwad and Solapur), henceforth referred to as high intensity and low intensity districts, respectively. Intensity of community mobilization efforts was assessed *a priori*, but is consistent with the descriptive results. Clustering at the site level using mixed models was not found to alter the strength or direction of significant associations between primary predictors and outcomes in the results, and therefore the models include district as a covariate among the other socio-demographic and sex-work practice variables.

### Ethical considerations

This study received ethical approval from the Institutional Ethical Review Board at St. John’s Medical College and Hospital, Bangalore, India, and the University of Manitoba Health Research Ethics Board, Winnipeg, Canada. Informed consent was obtained from all respondents prior to data collection.

## Results

### Characteristics of the study population

Overall, 1,750 female sex workers were surveyed in the five districts covered by the BTS. Table 2 summarizes their baseline characteristics. The mean age was 32, which varied by district (p < 0.001). The proportion of 18–21 year olds was three-fold higher in Solapur than in the other districts (p < .001). Overall, under half of the women had a main partner who was a husband compared to a non-cohabiting boyfriend (23%) or cohabiting partner (15%), though this was not the case in Solapur where nearly half of the women’s main partner was a boyfriend (p < 0.001). Just below 10% had initiated sex work before the age of 18 on average. Duration of sex work differed significantly by district (p < 0.001), with Dharwad having almost twice as many women who had been in sex work less than two years despite a similar mean age to other districts. In Solapur there was the most brothel-based and other more transient forms of sex work (p < .001), highest proportion (60% vs. less than 10% in other districts) of client volume more than 10 per week (p < .001), and greatest proportion of women to report having more than 10 sex partners per week (68% versus less than 10% in other districts, p < .001). These differences by district in socio-demographic and sex work practice variables seem to suggest that the women in Solapur, and to some extent also Dharwad, are more vulnerable overall.

In Solapur and Dharwad there was also lower community mobilization program intensity, with 62% and 71% of FSWs respectively reporting membership in a community-based organization (CBO), compared to more than 80% in the high intensity districts (p < .001). Duration of contact in Dharwad and Solapur was also lower than in other districts (p < .001). More FSWs in Solapur and Dharwad reported violence, coerced sex, and arrest than in Belgaum, Gulbarga and Gadag (p < .001 for each variable). Fewer women in Solapur and Dharwad reported always using condoms with clients and regular partners (p < .001 for both).

### Relationship between exposure variables and domains of power

Table 3 summarizes the findings of the multivariate analysis of the relationships between *power with*, *power within* and *power over* and socio-demographic, sex work, and program intervention variables. All *empowerment* means were lower in the low intensity districts (Solapur and Dharwad). Younger women (18–21) in all districts had lower average scores in all three empowerment domains than the oldest group, but this was only significant after adjustment in Dharwad and Solapur. Compared to the other sex-work typologies, women who solicited by telephone or who were brothel-based had significantly higher *power within*. In the high intensity districts, *power with others* was significantly lower among home-based and brothel-based sex workers. Women with more than five clients in the last week had significantly lower *power within* in all districts and lower *power with* in high intensity districts. Membership in a collective was associated with *power with*, remaining significant for women in low intensity areas. Time since first visited the program and higher contact with the program were associated with *power within* in the high intensity districts, and *power with* in all districts. In this analysis, *power over* resources was not significantly associated with program contact.

### Relationship between domains of power and health and social outcomes

Table 4 shows the associations between domains of power and self-efficacy, autonomy, violence and HIV risk, after adjustment. Data are shown for all women, and are disaggregated by intensity of community mobilization. The odds of having a high versus low autonomy level and never versus ever experiencing violence were 1.22 and 1.43 times higher respectively for every one unit increase in *power within* level in the high-intensity districts. *Power within* levels were also significantly positively associated with the odds of high self-efficacy for service utilization as well as actual service utilization in low intensity districts, and with the odds of high condom use with regular partners in high intensity districts. *Power with* was associated with higher odds of high self-efficacy for and actual condom use with clients in all districts. This finding was similar after disaggregation by intensity of community mobilization. An increase in *power over* levels was associated with higher odds of low condom use with regular clients in low intensity districts.

## Discussion

Our analysis of a large and representative survey of female sex workers in south India suggests that there are associations between community mobilization, reported levels of individual and collective empowerment, and improved health and social outcomes. These findings provide further evidence to support the hypothesis that community mobilization strategies promote social transformation and HIV risk reduction through the empowerment of FSWs.

We found an association between the amount and duration of exposure to community mobilization and greater empowerment levels, in particular *power with* and *power within*. This is consistent with findings in another study by KHPT assessing the benefits of membership in an FSW community-based organization, in which FSWs in focus-group discussions expressed both a sense of *power within* and *power with others* that they gained from membership [Bhattacharjee P, Prakash R, Pillai P, Isac S, Haranahalli M, Blanchard AK, Shahmanesh M, Blanchard JF, Moses S: WORLD BANK Special Issue: Understanding the role of peer group membership in reducing HIV-related risk and vulnerability among female sex workers in Karnataka, in preparation]. Although empowerment has not previously been measured using a comparable conceptual framework, these results are also consistent with findings from the *Sonagachi* project in Kolkata, in northeast India, where similar empowerment strategies were associated with an increased collective identity and social support (*power with*) and improved self-esteem and hope (*power within) *[14]. In Andhra Pradesh, South India, there was also a positive association found between exposure to an HIV prevention program and various measures of empowerment, including elements of collective identity (*power with*) and agency (*power to*) [8]. The fact that *power within* was significantly associated with program intervention variables in only Belgaum, Gulbarga and Gadag, while *power with* was associated with program interventions in all districts, may suggest that the community mobilization programs positively impact *power with* more quickly than *power within*. In our study, *power over resources* was not significantly associated with program exposure after adjusting for other factors. This contrasts with previous studies in Karnataka and Andhra Pradesh that have found that FSWs’ involvement with community mobilization led to economic independence (or *power over resources) *[8]. It may be that in our study, *power over resources* was not measured broadly enough; for example, we did not ask about strategies such as the use of micro-credit schemes.

There have been few previous studies that have measured direct associations between empowerment and outcomes such as social transformation, personal transformation in terms of self-efficacy, and proximate measures of HIV risk (service utilization and condom use). A study to assess the transformative effects of the *Sonagachi* Project’s initiatives to support collectivization of FSWs found that women with greater collective agency (*power to*) not only used condoms more consistently, but also benefited from changes in broader structural factors, such as personal safety and economic autonomy [18]. This is consistent with our findings of a significant association between collective empowerment (*power with*) and measures of social and personal transformation, including increased autonomy, reduced violence, and increased self-efficacy, even after adjustment for all other factors. In another study by KHPT, FSWs who were members of a peer-run community-based organization reported experiencing significantly less violence and were less likely to report having been forced to have sex [Bhattacharjee P, Prakash R, Pillai P, Isac S, Haranahalli M, Blanchard AK, Shahmanesh M, Blanchard JF, Moses S: WORLD BANK Special Issue: Understanding the role of peer group membership in reducing HIV-related risk and vulnerability among female sex workers in Karnataka, in preparation]. Women also expressed in focus-group discussions that group membership increased their ability to resist abuse from others in the community [Bhattacharjee P, Prakash R, Pillai P, Isac S, Haranahalli M, Blanchard AK, Shahmanesh M, Blanchard JF, Moses S: WORLD BANK Special Issue: Understanding the role of peer group membership in reducing HIV-related risk and vulnerability among female sex workers in Karnataka, in preparation].

From our surveys in five different south Indian districts, it appears that *power with* and *power within* were important for both improved self-efficacy for condom use and service utilization, and actual condom use and service utilization. Self-efficacy has been used as an indicator of a woman’s sense of *power to* address HIV vulnerability, while actual condom use and service utilization reflect reduced risk in our conceptual model. This confirms previous studies that have found associations between community mobilization and empowerment strategies and reduced HIV risk [8,14,16-18]. A previous study among FSWs in Andhra Pradesh measured collective power in terms of collective identity (similar to *power within)*, collective efficacy *(power with)*, and collective agency *(power to)*, and as in this study, collective identity and particularly collective agency seemed to be associated with consistent condom use [8]. Similarly, studies have also found that the empowerment strategies of the *Sonagachi* Project were associated with consistently improved condom negotiation and knowledge about STIs, related to improved collective identity and social support [14]. In a recent study by KHPT, members of a collective in focus group discussions also articulated that support from other FSWs (*power with*) improved their ability to negotiate condom use [Bhattacharjee P, Prakash R, Pillai P, Isac S, Haranahalli M, Blanchard AK, Shahmanesh M, Blanchard JF, Moses S: WORLD BANK Special Issue: Understanding the role of peer group membership in reducing HIV-related risk and vulnerability among female sex workers in Karnataka, in preparation].

The lack of consistent associations between *power over resources* and social outcomes may be due to the inadequacy of the questions asked in our survey, as suggested above, or because access to social entitlements was very high across all participants. Other studies have found access to credit and entitlements to improve women’s ability to reduce their HIV vulnerability, as well as supporting their sense of *power within* and the strength of CBOs [8,14,28,31]. Qualitative studies conducted by KHPT suggest that access to microcredit schemes strengthened women’s ability to negotiate, and specifically to refuse unprotected sex [28], [Bhattacharjee P, Prakash R, Pillai P, Isac S, Haranahalli M, Blanchard AK, Shahmanesh M, Blanchard JF, Moses S: WORLD BANK Special Issue: Understanding the role of peer group membership in reducing HIV-related risk and vulnerability among female sex workers in Karnataka, in preparation].

This study has several limitations. First, the surveys were cross-sectional, so we cannot ascertain the direction of causality. For example, it could be that more empowered and lower risk FSWs were more likely to join a collective than their more high-risk and disempowered colleagues. Second, as there was no pre-identified control group, there is potential for participation bias, both in terms of the type of FSW who becomes “empowered” and the type of risk environment that facilitates empowerment. For example, it may be that the same factors that make Solapur less conducive to collectivization render it a higher risk environment for the FSWs who work there. Third, the responses were self-reported on an individual basis, even for variables measuring collective processes of empowerment. The resulting misclassification bias could be further exacerbated if the process of community engagement leads to social desirability in the responses of individual FSWs to questions on empowerment. Fourthly, CBO membership and program coverage, particularly at the time of the BTS, were very high. Moreover, there had already been extensive work done by KHPT to improve the risk environment. This could have diluted some of the perceived effects of community engagement, so that even those FSWs without direct involvement with CBOs or direct contact with the program would benefit from the indirect effects of structural changes to the risk environment.

## Conclusions

This study supports the hypothesis that community mobilization leads to greater empowerment of female sex workers, including *power with*, *power within* and *power over*. This construct is important for giving individual FSWs greater *power to* reduce their vulnerability, by improving their ability to access services and HIV prevention technologies, as well as transforming their risk environment. Conceptualizing empowerment in terms of increasing individual and collective power in the different power domains can serve as a flexible tool for prospectively tracking community mobilization in a systematic and reproducible way. It would be valuable to conduct prospective studies to examine the relative importance of different domains of empowerment; the effect of different interventions on domains of empowerment; the direction of effects; and the impact of empowerment on health outcomes such as access to HIV prevention technologies, the incidence of HIV infection and other sexually transmitted infections, and unwanted pregnancy. It will be important to continue generating evidence in relation to addressing the societal factors underlying FSWs’ HIV vulnerability in a variety of contexts.

## Competing interests

The authors declare that they have no competing interests.

## Authors’ contributions

HLM, SI, BMR, PB, VG, SM, and JFB were involved in implementation of the intervention, designing and supervising the data collection and analysis, and interpretation of the findings. AKB helped to compile the data and conducted the data analysis, and MS synthesized the findings and drafted a report on the findings for the World Bank. All authors contributed to writing this paper. All authors read and approved the final manuscript.

## Pre-publication history

The pre-publication history for this paper can be accessed here:

http://www.biomedcentral.com/1471-2458/13/234/prepub

## References

[B1] Blanchard JF Halli S Ramesh BM Bhattacharjee P Washington RG O'Neil J Moses S **Variability in the sexual structure in a rural Indian setting: implications for HIV prevention strategies** Sex Transm Infect 2007 83 Suppl 1 i30 36 17664362 10.1136/sti.2006.023572

[B2] Blanchard JF O'Neil J Ramesh BM Bhattacharjee P Orchard T Moses S **Understanding the social and cultural contexts of female sex workers in Karnataka, India: implications for prevention of HIV infection** J Infect Dis 2005 191 Suppl 1 S139 146 15627224 10.1086/425273

[B3] Campbell C **Selling sex in the time of AIDS: the psycho-social context of condom use by sex workers on a Southern African mine** Soc Sci Med 2000 50 4 479 494 10.1016/S0277-9536(99)00317-2 10641801

[B4] O'Neil J Orchard T Swarankar RC Blanchard JFJF Gurav K Moses S **Dhandha, dharma and disease: traditional sex work and HIV/AIDS in rural India** Soc Sci Med 2004 59 4 851 860 10.1016/j.socscimed.2003.11.032 15177840

[B5] Wight D Plummer ML Mshana G Wamoyi J Shigongo ZS Ross DA **Contradictory sexual norms and expectations for young people in rural Northern Tanzania** Soc Sci Med 2006 62 4 987 997 10.1016/j.socscimed.2005.06.052 16139937

[B6] Amin A Overs C Saunders P **Violence against sex workers and HIV prevention** Violence Against Women and HIV/AIDS: Critical Intersections 2005 Geneva: World Health Organization 1 6

[B7] Parker R Aggleton P RaA P **HIV- and AIDS-related Stigma and Discrimination: a conceptual framework and implications for action** Culture, Society and Sexuality: a Reader 2007 2 London: Routeledge 10.1016/s0277-9536(02)00304-0 12753813

[B8] Blankenship KM West BS Kershaw TS Biradavolu MR **Power, community mobilization, and condom use practices among female sex workers in Andhra Pradesh, India** AIDS 2008 22 Suppl 5 S109 116 10.1097/01.aids.0000343769.92949.dd 19098471

[B9] Halli SS Ramesh BM O'Neil J Moses S Blanchard JF **The role of collectives in STI and HIV/AIDS prevention among female sex workers in Karnataka** India. AIDS Care 2006 18 7 739 749 10.1080/09540120500466937 16971283

[B10] Moses S Ramesh BM Nagelkerke NJ Khera A Isac S Bhattacharjee P Gurnani V Washington R Prakash KH Pradeep BS **Impact of an intensive HIV prevention programme for female sex workers on HIV prevalence among antenatal clinic attenders in Karnataka state, south India: an ecological analysis** AIDS 2008 22 Suppl 5 S101 108 10.1097/01.aids.0000343768.85325.92 19098470

[B11] Evans C Jana S Lambert H **What makes a structural intervention? Reducing vulnerability to HIV in community settings, with particular reference to sex work** Glob Public Health 2010 5 5 449 461 10.1080/17441690902942472 19507079

[B12] Cornish F **Empowerment to participate: a case study of participation by indian sex workers in HIV prevention** Journal of Community & Applied Social Psychology 2006 16 4 301 315 10.1002/casp.86623560319

[B13] Kabeer N **Resources, Agency, Achievements: Reflections on the Measurement of Women's Empowerment** Development and Change 1999 30 435 464 10.1111/1467-7660.00125

[B14] Swendeman D Basu I Das S Jana S Rotheram-Borus MJ **Empowering sex workers in India to reduce vulnerability to HIV and sexually transmitted diseases** Soc Sci Med 2009 69 8 1157 1166 10.1016/j.socscimed.2009.07.035 19716639 PMC2824563

[B15] Kim JC Watts CH Hargreaves JR Ndhlovu LX Phetla G Morison LA Busza J Porter JD Pronyk P **Understanding the impact of a microfinance-based intervention on women's empowerment and the reduction of intimate partner violence in South Africa** Am J Public Health 2007 97 10 1794 1802 10.2105/AJPH.2006.095521 17761566 PMC1994170

[B16] Reza-Paul S Beattie T Syed HU Venukumar KT Venugopal MS Fathima MP Raghavendra HR Akram P Manjula R Lakshmi M **Declines in risk behaviour and sexually transmitted infection prevalence following a community-led HIV preventive intervention among female sex workers in Mysore, India** AIDS 2008 22 Suppl 5 S91 100 10.1097/01.aids.0000343767.08197.18 19098483

[B17] Basu I Jana S Rotheram-Borus MJ Swendeman D Lee SJ Newman P Weiss R **HIV prevention among sex workers in India** J Acquir Immune Defic Syndr 2004 36 3 845 852 10.1097/00126334-200407010-00012 15213569 PMC2826108

[B18] Ghose T Swendeman Dallas George Sheba Chowdhury Debasish **Mobilizing collective identity to reduce HIV risk among sex workers in Sonagachi, India: The boundaries, consciousness, negotiation framework** Soc Sci Med 2008 67 2 311 320 10.1016/j.socscimed.2008.03.045 18455855 PMC2881846

[B19] Busza J Schunter BT **From competition to community: participatory learning and action among young, debt-bonded Vietnamese sex workers in Cambodia** Reprod Health Matters 2001 9 17 72 81 10.1016/S0968-8080(01)90010-2 11468849

[B20] Kabeer N **Gender Equality, Poverty Eradication and the Millennium Development Goals: Promoting Women's Capabilities and Participation** Women in Development Discussion Paper Serieas 2003 Sussex: Institute of Development Studies 1 26

[B21] Kabeer N **Gender equality and women's empowerment: a critical analysis of the third Millennium Development Goal** Gender Mainstreaming in Poverty Eradication and the Millennium Development Goals: A Handbook for Policy-makers and Other Stakeholders 2003 London: Commonwealth Secretariat 13 24 edn

[B22] Sen A **Women's Agency and Social Change** Development as Freedom 1999 New York: Anchor Books 188 203 edn

[B23] Cornish F Ghosh R **The necessary contradictions of 'community-led' health promotion: a case study of HIV prevention in an Indian red light district** Soc Sci Med 2007 64 2 496 507 10.1016/j.socscimed.2006.09.009 17055635

[B24] Cornish F Shukla A Banerji R **Persuading, protesting and exchanging favours: strategies used by Indian sex workers to win local support for their HIV prevention programmes** AIDS Care 2010 22 Suppl 2 1670 1678 21161773 10.1080/09540121.2010.521545

[B25] Asthana S Oostvogels R **Community participation in HIV prevention: problems and prospects for community-based strategies among female sex workers in Madras** Soc Sci Med 1996 43 2 133 148 10.1016/0277-9536(95)00348-7 8844919

[B26] Rahman A People's Self-Development 1993 Dhaka: The University Press Limited

[B27] Campbell C Cornish F How Can Community Health Programmes Build Enabling Environments for Transformative Communication? 2011 AIDS Behav: Experiences from India and South Africa 10.1007/s10461-011-9966-2 21604108

[B28] Pillai P Bhattacharjee P Ramesh BM Isac S Impact of two vulnerability reduction strategies - collectivization anad participation in savings activities - on risk reduction among FSWs 2011 Bangalore: Karnataka Health Promotion Trust 1 110

[B29] Panchanadeswaran S Johnson SC Sivaram S Srikrishnan AK Latkin C Bentley ME Solomon S Go VF Celentano D **Intimate partner violence is as important as client violence in increasing street-based female sex workers' vulnerability to HIV in India** Int J Drug Policy 2008 19 2 106 112 10.1016/j.drugpo.2007.11.013 18187314 PMC2423812

[B30] Wojcicki JM Malala J **Condom use, power and HIV/AIDS risk: sex-workers bargain for survival in Hillbrow/Joubert Park/Berea, Johannesburg** Soc Sci Med 2001 53 99 121 10.1016/S0277-9536(00)00315-4 11380165

[B31] Reed E Gupta J Biradavolu M Devireddy V Blankenship KM **The context of economic insecurity and its relation to violence and risk factors for HIV among female sex workers in Andhra Pradesh, India** Public Health Rep 2010 125 Suppl 4 81 89 20629253 10.1177/00333549101250S412 PMC2882978

[B32] Bourcier E Girma M Downer A Douglas E Gonzales V Weaver M Douglas E Washienko K Levine R Yamamoto I Huckeba H Katai A Project TS **Room for Change: Preventing HIV Transmission in Brothels** Transmission Settings, Part III-Brothels 2002 Seattle: University of Washington Center for Health Education and Research

[B33] Kerrigan D Ellen JM Moreno L Rosario S Katz J Celentano DD Sweat M **Environmental-structural factors significantly associated with consistent condom use among female sex workers in the Dominican Republic** AIDS 2003 17 3 415 423 10.1097/00002030-200302140-00016 12556696

[B34] Kerrigan D Moreno L Rosario S Gomez B Jerez H Barrington C Weiss E Sweat M **Environmental-structural interventions to reduce HIV/STI risk among female sex workers in the Dominican Republic** Am J Public Health 2006 96 1 120 125 10.2105/AJPH.2004.042200 16317215 PMC1470438

[B35] Tucker JD Tuminez AS **Reframing the interpretation of sex worker health: a behavioral-structural approach** J Infect Dis 2011 204 Suppl 5 S1206 1210 10.1093/infdis/jir534 22043033 PMC3205084

[B36] Gurnani V Beattie TS Bhattacharjee P Mohan H Maddur S Washington R Isac S Ramesh B Moses S Blanchard JF **An integrated structural intervention to reduce vulnerability to HIV and sexually transmitted infections among female sex workers in Karnataka state, south India** BMC Public Health 2011 11 755 10.1186/1471-2458-11-755 21962115 PMC3205062

[B37] Dunteman GH Principal Components Analysis 1989 Thousand Oaks, California: Sage University Press 1 97 In. vol. 69.

[B38] Jolliffe IT **Principal Component Analysis** Encyclopedia of Statistics in Behavioural Science 2005 Malden Michigan: John Wiley and Sons Ltd

